# Non-surgical Approach to a Maxillary Cyst-Like Lesion: Orthograde Endodontic Treatment With Neodymium-Doped Yttrium Aluminum Garnet (Nd:YAG) Decontamination of the Canal System

**DOI:** 10.7759/cureus.90866

**Published:** 2025-08-24

**Authors:** Beatrice Spaggiari, Paolo Vescovi, Silvia Pizzi, Roberta Iaria, Ilaria Giovannacci

**Affiliations:** 1 Department of Medicine and Surgery, University of Parma, Parma, ITA

**Keywords:** endodontic treatment, laser assisted decontamination, nd:yag laser, nonsurgical treatment, radicular cyst-like lesion

## Abstract

The aim of this study is to describe the nonsurgical resolution of a large periapical cyst-like lesion through traditional endodontic treatment, supported by neodymium-doped yttrium aluminum garnet (Nd:YAG) laser activation of the irrigants. A 16-year-old girl was referred to our service with swelling of the right hemiface, pus, pain, and intraoral swelling of the labial mucosa extending from the right upper incisor (element 11) to the right upper canine (element 13). The radiological examination, a cone beam computed tomographic scan provided at the first appointment, confirmed the presence of a large cyst-like lesion extending from the right upper central incisor to the right upper canine. The right upper central and lateral incisors underwent endodontic treatment with the support of an Nd:YAG laser to enhance the decontamination action of the irrigants. The one-year follow-up showed complete healing of the lesion. This successful outcome supports the potential for healing of cyst-like lesions through nonsurgical treatment. However, the role of the Nd:YAG laser in endodontics requires further study.

## Introduction

Typically, inflammatory periapical lesions of endodontic origin range from 5 to 8 mm in diameter. Lesions up to 10 mm are classified as granulomas, whereas larger ones are classified as apical cysts [1].

Periapical cysts are inflammatory and the most common odontogenic cysts. The anterior region of the maxilla is the area most commonly affected [2]. These cysts develop at the root apex of a nonvital tooth because of inflammation caused by dental caries or trauma. This inflammation activates and promotes the proliferation of the epithelial cell rests of Malassez located around the apex of the affected tooth. As a result, osmotic pressure increases, causing the cyst’s expansion. Nair et al.'s study of 256 periapical lesions obtained from extracted teeth identified two distinct classes of radicular cysts: the apical true cyst and the pocket cyst (‘bay’ cysts) [3].

A periapical true cyst is defined as an epithelium-lined, closed pathological cavity at the apex of a tooth. Although the pathogenesis has been widely discussed, the apical cyst is now considered a direct evolution of a granuloma. However, not all granulomas necessarily develop into apical cysts [4]. According to Toller, the exact mechanism of cyst growth remains unknown. It is generally believed that the presence of necrotic tissue within the cyst lumen attracts neutrophilic granulocytes, which die after migrating through the epithelial lining. These dying cells produce numerous molecules, resulting in an increase in osmotic pressure [5].

The periapical pocket cyst is a radicular cyst with an epithelium-lined cavity that communicates directly with the root canal system through the apical foramen, with the apex protruding into the cavity [6]. Although the clinical implications of this distinction are significant, clinicians cannot determine the histological nature of a radiolucent lesion during treatment. 

Extensive periapical lesions may not always heal, and surgical interventions may be necessary. The primary goal of traditional root canal treatment is to eliminate infectious agents from the canal system and prevent reinfection through obturation. Periapical pocket cysts are more likely to heal, as published case reports have shown [7]. In contrast, true cysts are considered self-sustaining because the lesion no longer depends on the root canal and thus is less likely to be resolved by conventional endodontic treatment [4].

Endodontic options for the management of radicular cysts

The American Association of Endodontists recommends that apical surgery be considered only in cases that cannot be treated otherwise. Therefore, the primary approach used today is orthograde endodontic treatment (with a success rate of 85-90%), whereas surgery is a secondary option. During traditional orthograde endodontic therapy, instrumentation alone is insufficient to achieve healing of a periapical infection; therefore, the irrigation process is an essential step [8].

The most commonly used irrigants in endodontics are sodium hypochlorite (NaOCl) and ethylenediaminetetraacetic acid (EDTA). NaOCl is an alkaline solution with bactericidal and organic tissue-dissolving properties. Its clinical concentration ranges from 0.5% to 8%. Increasing the concentration enhances both its bactericidal effect and tissue degradation ability [9]. However, causticity and cytotoxicity also increase proportionally. EDTA is a calcium chelator that removes the smear layer and debris from the root canal walls. It is used in combination with NaOCl because of its ability to open dentinal tubules, allowing NaOCl to act more deeply [9]. EDTA is usually applied in a 17% solution. Another possible formulation is a 10% gel, which helps reduce friction during the shaping phase of the canal, at the expense of reduced visibility. It should never be used as the final irrigant, because it softens the root canal walls.

The inoculation of the irrigants typically occurs by a conventional syringe with very small diameter needles (30 gauges). However, many factors may limit the efficacy of syringe irrigation, such as the width, curvature, and taper of the tooth canal [9]. Because of these limitations, the need to enhance the effects of irrigants has been investigated over the years, and the use of laser technologies in endodontics is an interesting step forward in decontamination procedures.

Several laser technologies can be applied to decontaminate the root canal system, including near-infrared (NIR) wavelengths (810-1064 nm), photodynamic antimicrobial disinfection (aPDT), and mid-infrared wavelengths (2780-2940 nm). NIR wavelengths include diode lasers (810-980 nm) and neodymium-doped yttrium aluminum garnet (Nd:YAG) lasers (1064 nm). They interact with the host tissue through a photothermal effect. Their penetration into dentin can exceed 1000 µm through scattering and transmission along the dentinal tubules [10]. These lasers are absorbed by chromophores; therefore, only pigmented microorganisms are directly inactivated. However, an additional microbicidal effect results from photothermal damage [11]. Moreover, several studies have shown that Nd:YAG lasers remove debris and the smear layer from the root canal walls, improving decontamination of the endodontic system [12].

Koba et al. investigated the role of Nd:YAG lasers in post-operative symptoms and healing of infected teeth after endodontic treatment. The results suggested that the application of pulsed Nd:YAG lasers (1.5 W, 15Hz, four times for 5 sec) might be advantageous for managing infected root canals [13,14].

NIR wavelengths can be used inside the canal with or without an irrigant solution. This application of NIR wavelengths has been defined as “traditional laser endodontics” [15]. Diode lasers, which fall within the NIR wavelength range, can be used in aPDT, a type of disinfection that requires a photosensitizer to be injected inside the canal. The photosensitizer is a compound that, when irradiated by an appropriate light source, responds by releasing oxygen free radicals and singlet oxygen. These act directly on the cellular structures of microbial cells, without inducing cytotoxicity in the host cells. The photosensitizers most commonly described in endodontics are methylene blue, toluidine blue, indocyanine green, and curcumin [16]. Literature describes the possibility of pigmentation of the treated tooth due to the photosensitizer, so this eventuality must be taken into account when choosing a laser disinfection strategy (for example, if treating an aesthetic area) [17].

Obtaining an optimal apical seal is essential to improve the chances of lesion healing. In cases where a natural apical constriction is absent, strategies can help compact the filling materials while avoiding extrusion beyond the apex [18]. For example, a platelet-rich fibrin matrix or a collagen sponge can be applied [19]. This report describes the resolution of a large periapical cyst lesion after a traditional orthograde root canal treatment, supported by Nd:YAG laser activation of 5% NaOCl for the decontamination of the root canal system.

## Case presentation

A 16-year-old girl was referred by a colleague for the management of a maxillary periapical cyst-like lesion involving the right upper central incisor, lateral incisor, and canine. The patient, accompanied by her mother, reported an episode of acute facial swelling for which she went to the local ER. There, after evaluation with cone beam computed tomography (CBCT), antibiotic therapy was administered.

A colleague performed emergency drainage by opening the pulp chamber of tooth 11 and referred the patient to us for evaluation. In her medical history, the patient reported facial trauma at the age of eight, resulting in coronal trauma (Class II trauma, according to Ellis' classification, with enamel and dentin fracture without pulp exposure) [20] to the right upper central incisor (11) and transient mobility of the right upper lateral incisor (13).

**Figure 1:**
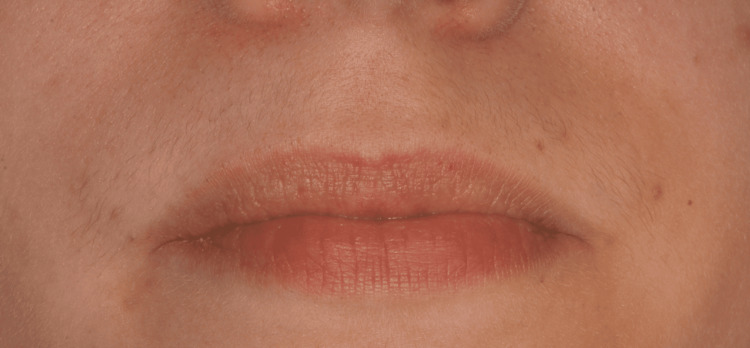
Swelling of the right hemiface, resulting in facial asymmetry

Extraoral clinical examination revealed facial swelling with evident asymmetry of the right hemiface (Figure 1).

Intraoral clinical examination revealed pus and painful swelling of the labial mucosa, extending from the right upper central incisor to the right upper canine. Overall, the intraoral swelling extended for approximately two centimeters, starting from tooth 11 and extending to tooth 13. On palpation, the swelling felt soft and fluctuant at teeth 11 and 12, while it was hard and immobile at tooth 13, where pus was most noticeable.

**Figure 2:**
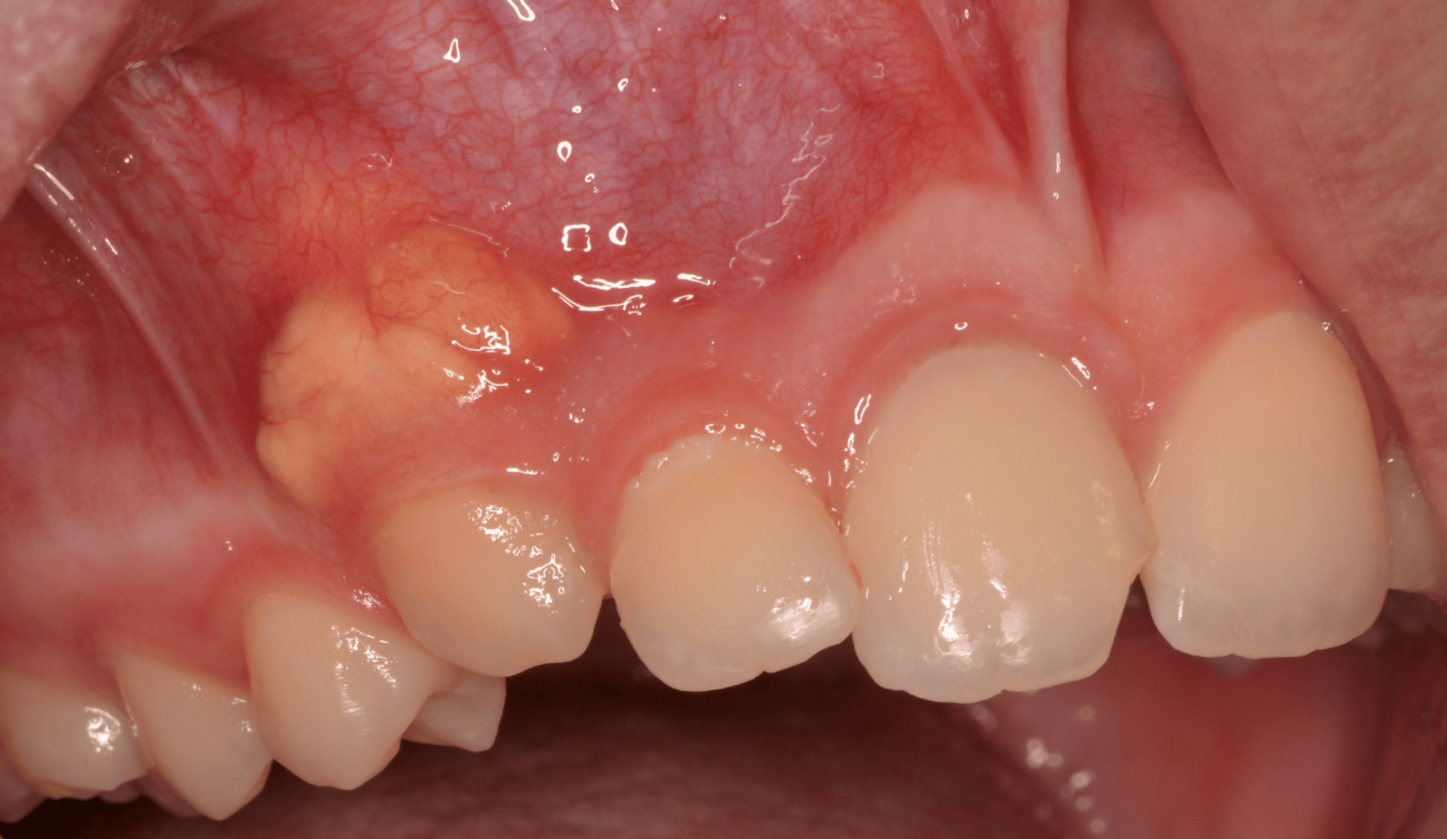
Intraoral manifestation of the lesion with pus, swelling, and pain of the labial mucosa

The swelling of the right side of the face, extending from the lower right eyelid to the upper right lip, confirmed the presence of a sinus tract, with no signs of decay or periodontal pockets (Figure 2).

Thermal sensitivity tests (cold test Crio Spray SZ 114; Karl Sanremo®, Italy) were positive from the right upper central incisor (11) to the first premolar (14), whereas adjacent teeth presented normal responses (elements 15, 16, 17). Crio Spray is an hypothermic agent based on tetrafluoroethane, with fine mint essence. The test was performed by applying a cotton pellet to the crowns of teeth 11 to 17. Any reaction (verbal or nonverbal, such as moving the head away from the cotton pellet) was considered a sign of vitality. For each tooth that did not respond, there was a maximum wait time of 10 seconds before recording the response. The right upper central and lateral incisors were also painful to vertical percussion (Table 1).

**Table 1:** Results of diagnostic tests at the first visit

Tooth	Cold	Percussion	Apical palpation	Mobility
Central incisor (11)	No response	Painful	Painful	Within normal limit
Lateral incisor (12)	No response	Painful	Painful	Within normal limit
Canine (13)	No response	Mild	Painful	Within normal limit
First premolar (14)	No response	Normal	Mild	Within normal limit
Second premolar (15)	Mild	Normal	Mild	Within normal limit
First molar (16)	Normal	Normal	Normal	Within normal limit
Second molar (17)	Normal	Normal	Normal	Within normal limit

**Figure 3:**
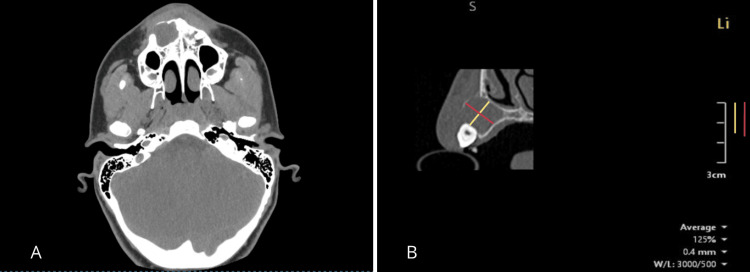
Cone beam computed tomography (CBCT) images A) Initial CBCT provided by the patient. The axial view shows the significant site of the lesion with cortical plate perforation. B) CBCT sagittal view with scale in cm. The lesion measures approximately 1.5 x 1.5 cm.

CBCT revealed a large (approximately 1.5 x 1.5 cm), unilocular, periapical radiolucency with well-defined margins, that had caused a buccal cortical plate perforation without involving the noble structures (axial view) (Figure 3).

**Figure 4:**
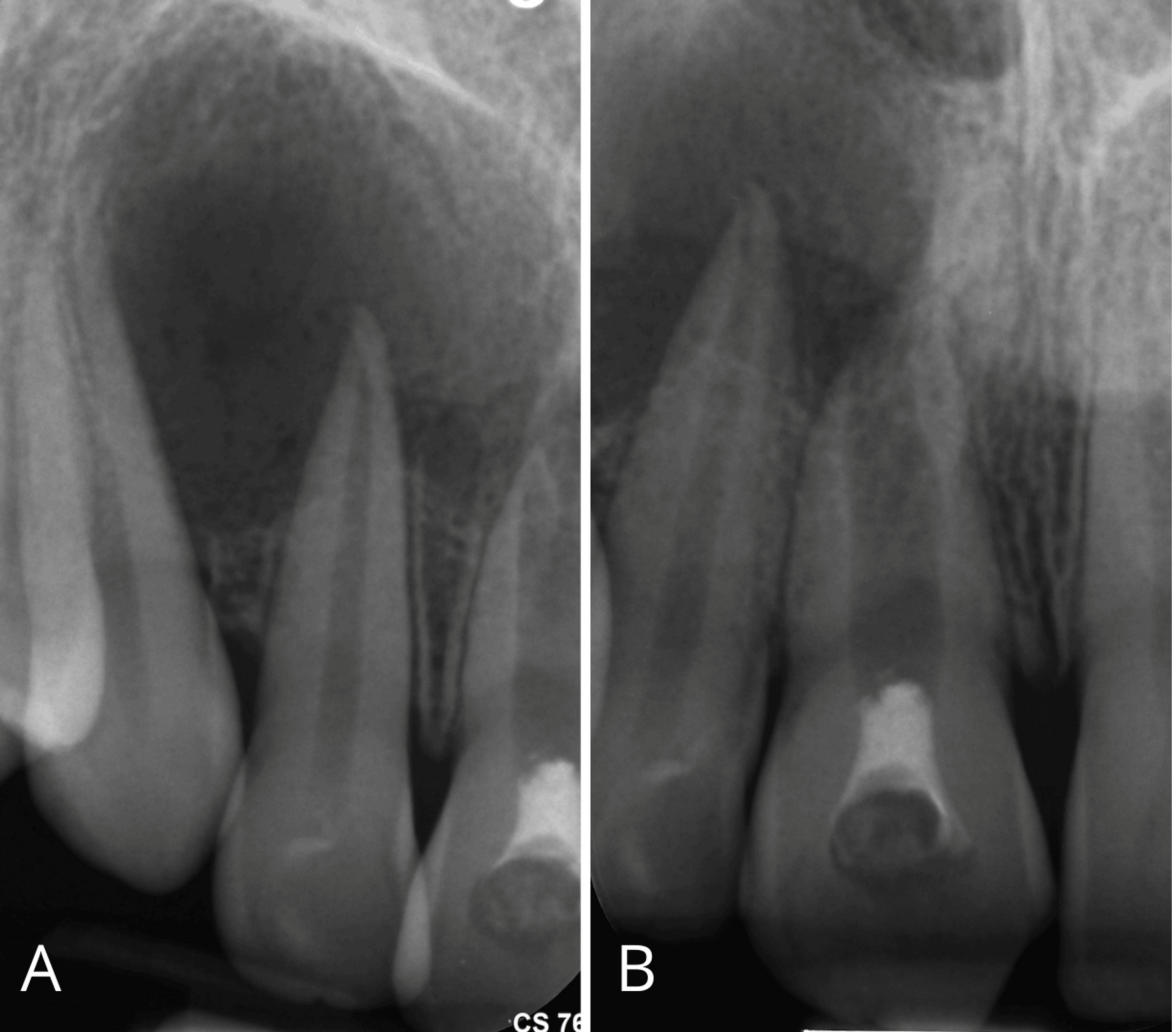
Intraoral X-ray images A) Intraoral x-rays taken after emergency drainage. The lesion extends from the distal portion of the upper central incisor to the canine. B) The central incisor shows a blunderbuss canal with an open apex.

Periapical radiographs showed a radiolucent area extending from the distal aspect of the upper central incisor, including the lateral incisor, to the mesial surface of the upper right canine. They also revealed the complex anatomy of the central incisor, with a large canal system and open apex (Figures 4a, 4b).

Based on the radiographic and clinical examinations, root canal treatment of the central incisor was performed. In literature, the success rate for conservative or surgical approaches is comparable (in both cases, the rates are around 90%) [21,22].The clinician must therefore determine the most appropriate procedure for each patient by assessing the localization of the lesion, its extension, its proximity to noble structures, and factors related to the patient (age, comorbidity). In this specific case, given the young age of the patient, a conservative approach was considered more prudent, with surgery as a secondary option in the event of incomplete healing. The patient’s mother agreed with the treatment plan and provided informed consent.

The pulp chamber of the central incisor was opened by a colleague in emergency. The pus was drained, followed by irrigation with 10 mL of saline. A calcium hydroxide intracanal medicament was placed for one month. At this point, there was no more drainage of fluids from the tooth, and endodontic treatment was performed.

The tooth was isolated with a rubber dam and irrigated again with saline. Electronic determination of working length (WL) was unreliable. When apical constriction is absent and no manual tactile resistance is felt, radiography becomes the primary method for determining WL.

Debridement and irrigation, both critical to the success of endodontic treatment, involve the risk of apical extrusion of debris and NaOCl, which can negatively affect apical healing. Therefore, soft debridement was performed using nickel-titanium rotary instrument (step-back technique, MTwo® (Sweden&Martina n.40.06, Padova, Italy), with soft irrigation using 5% NaOCl and 17% EDTA. The debridement procedure applied was very gentle, due to the already thin dentinal walls. Irrigants played a fundamental role in decontamination and, to this end, were applied for approximately 30 minutes, and refreshed approximately every minute.

**Figure 5:**
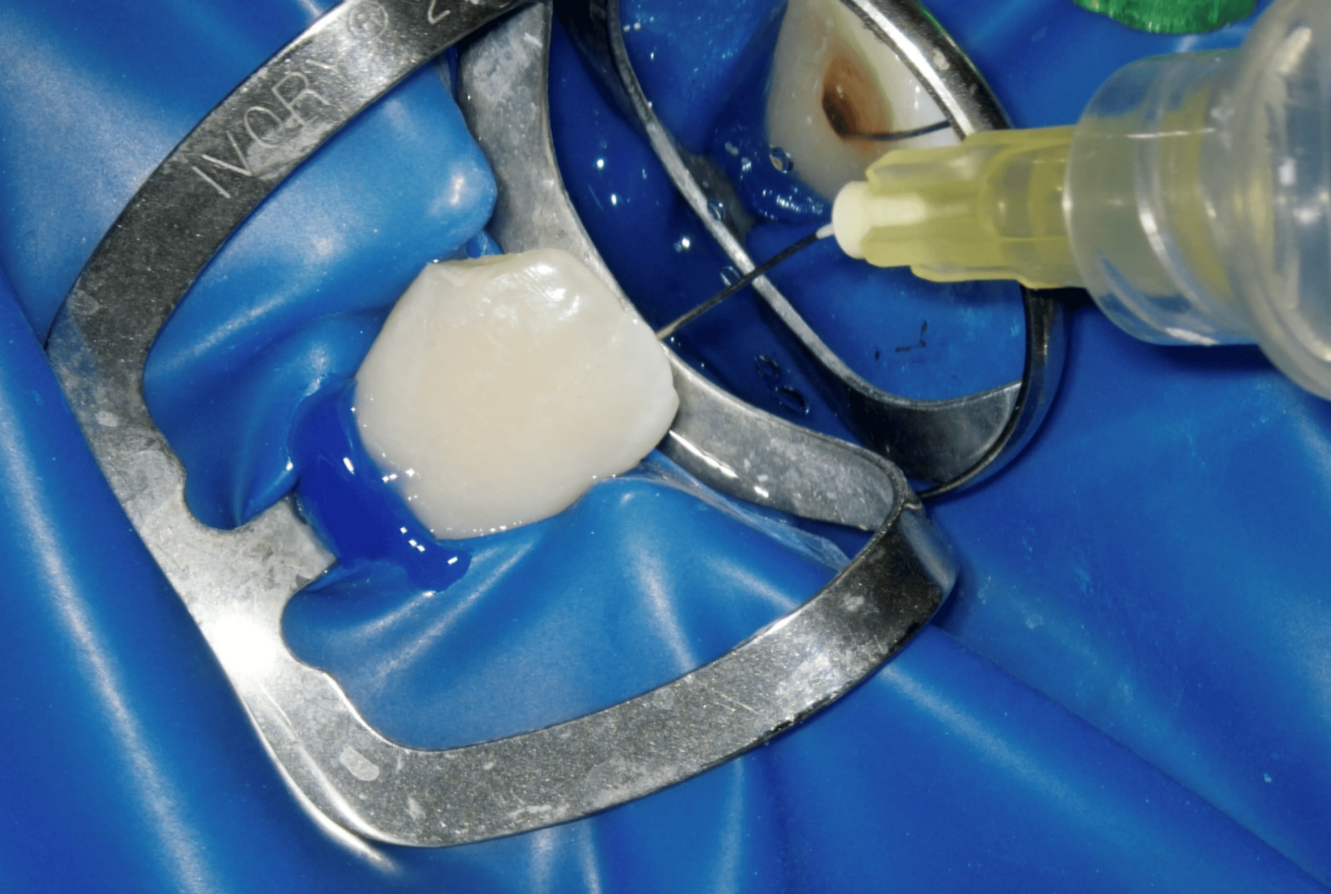
5% NaOCl activation with Nd:YAG laser (1.5 W, 15Hz, 200 nm fiber) NaOCl: sodium hypochlorite; Nd:YAG laser: Neodymium-doped yttrium aluminum garnet. Nd:YAG laser (LightWalker®, Fotona, Ljubljana, Slovenia)

In recent years, many studies have supported the use of laser as an effective tool to improve debris and smear layer removal. Its effectiveness also includes decontamination. Nd:YAG laser (15Hz, 1.5W; LightWalker®, Fotona, Ljubljana, Slovenia) was applied for the final NaOCl irrigation. A 200 µm fibre was inserted into a canal 5 mm shorter than the WL, and activated in a coronal direction. This procedure lasted for five seconds and was repeated four times (Figure 5) [15].

Once disinfection was complete, the canal was ready for obturation. In case of open apexes (e.g., apexes >60), standard obturation with gutta-percha using a continuous wave of condensation is not recommended because of the inability of achieving a proper apical plug.

**Figure 6:**
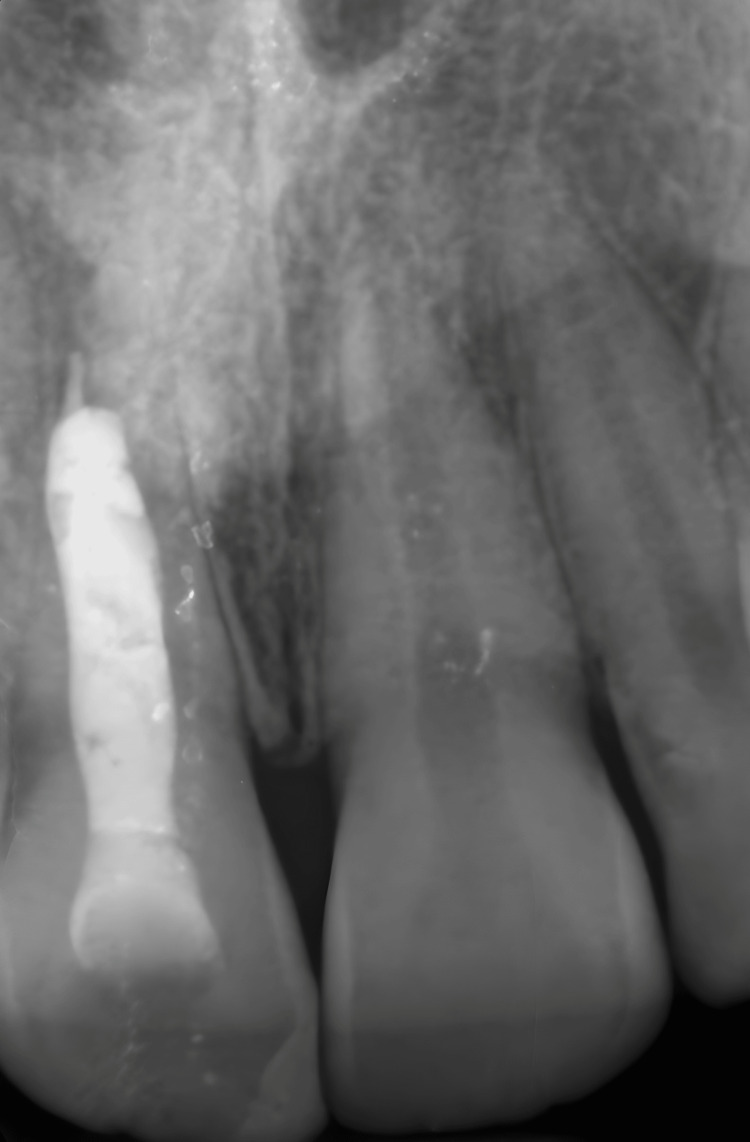
Final X-ray after endondontic treatment of the right central incisor The apical third root filling was with Super EBA™ (Harry J. Bosworth Co., Skokie, IL) and warm guttapercha was used for the backfilling.

After drying the root canal with a sterilized paper point, a collagen sponge fragment was introduced at the apex using a #3 gutta-percha condenser. Super EBA™ (Harry J. Bosworth Co., Skokie, IL) was placed in the apical third of the canal system at approximately 5 mm thickness using a special device for inserting material (Map System, Produits Dentaires SA, Switzerland). The remaining portion of the canal was obturated with warm gutta-percha (Figure 6).

**Figure 7:**
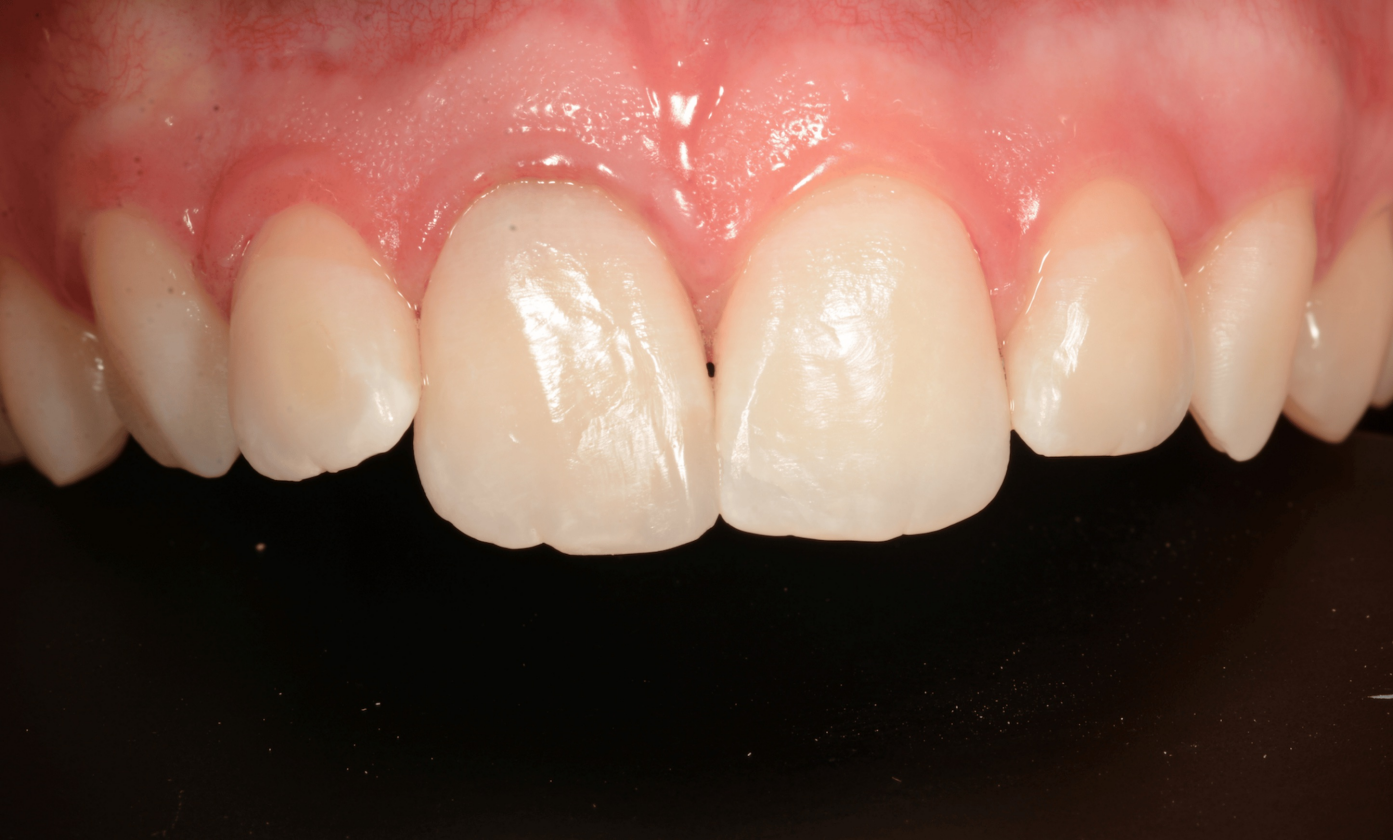
Recovery of the right central incisor with a direct restorative

At this point, the patient was ready for the restorative procedures (Figure 7).

After three months, pus and swelling in the labial mucosa was absent, and the right upper lateral incisor remained negative on thermal sensitivity testing and painful on vertical percussion. Consequently, endodontic treatment of the tooth was performed, following a shared decision-making process with the patient and her mother.

The lateral incisor presented a conventional anatomy, so standard procedures were implemented. After isolation with rubber dam, the pulp chamber was exposed and the canal orifice was located using sonic instruments (Kavo, Soniflex Endo). A K-file n°10 with a rubber stop was passively introduced into the root canal, and WL was electronically measured (Morita apex locator; J. Morita Corp., J. Morita, Europe).

**Figure 8:**
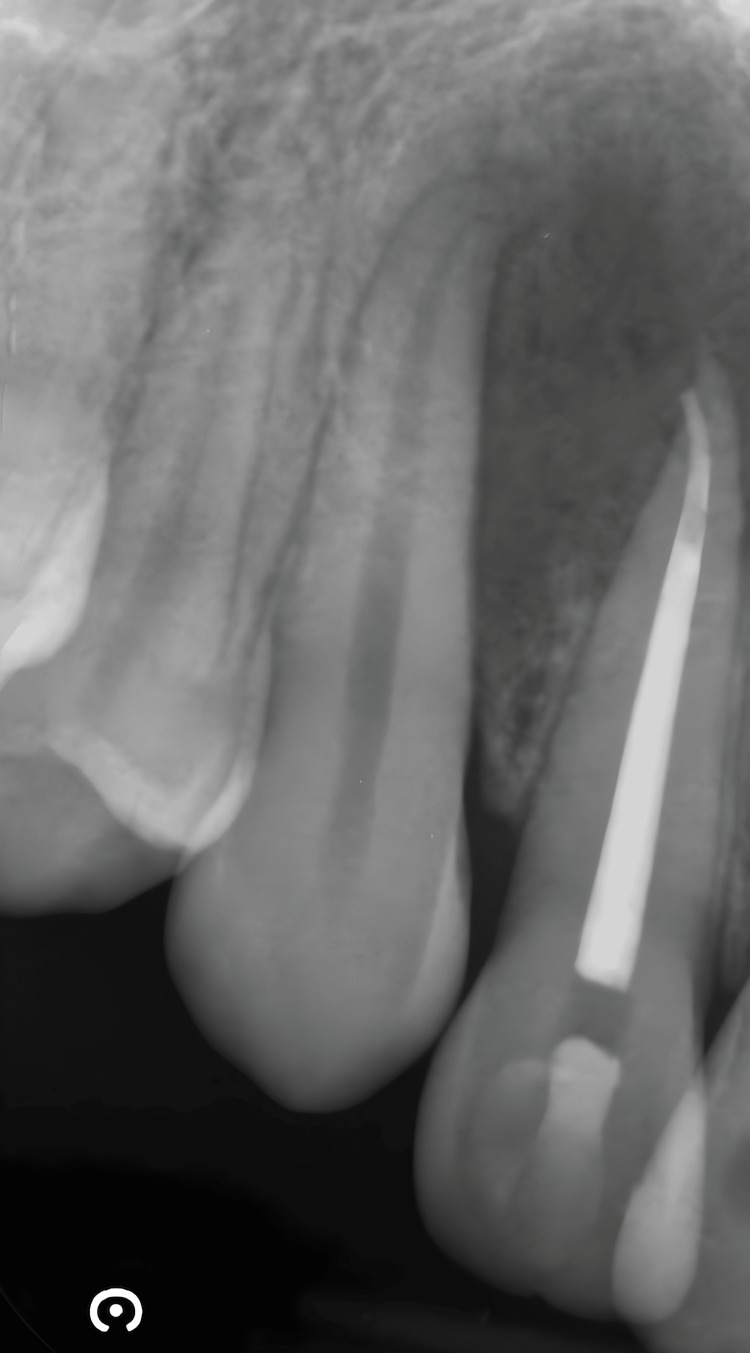
Endodontic treatment of the right upper later incisor (continuous wave of condensation technique)

Canal instrumentation was performed using nickel-titanium rotary instruments (MTwo Sweden&Martina from 10.04 to 25.06). Smear layer removal was achieved using irrigant solutions, specifically 5% NaOCl and 17% EDTA, applied in the final minutes of treatment. The final WL was electronically verified, and then the canal was irrigated again with 5% NaOCl and dried with sterilized paper point. Canal obturation with gutta-percha was obtained with continuous wave of condensation (Figure 8).

**Figure 9:**
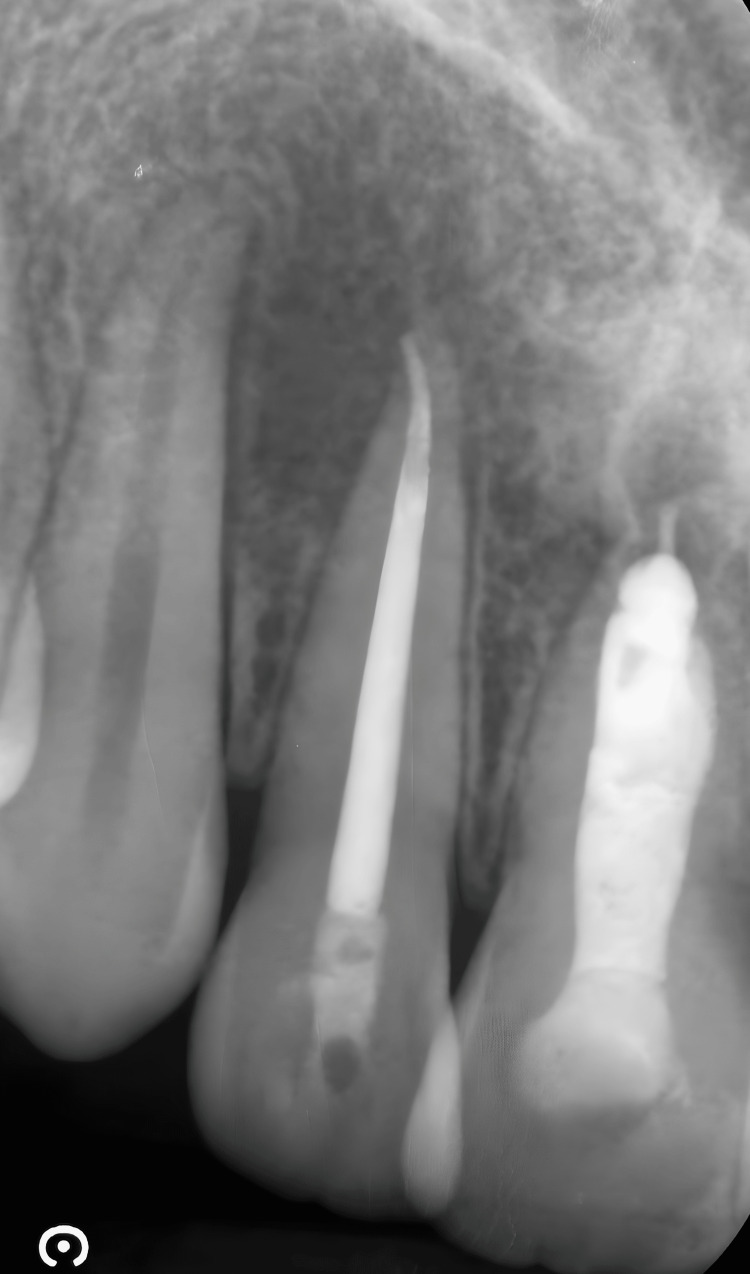
Intraoral X-ray at the six-month follow up visit showing the lesion's positive healing trend

At the six-month follow-up, the patient reported no symptoms, and radiographic control revealed a positive healing trend of the lesion (Figure 9).

**Figure 10:**
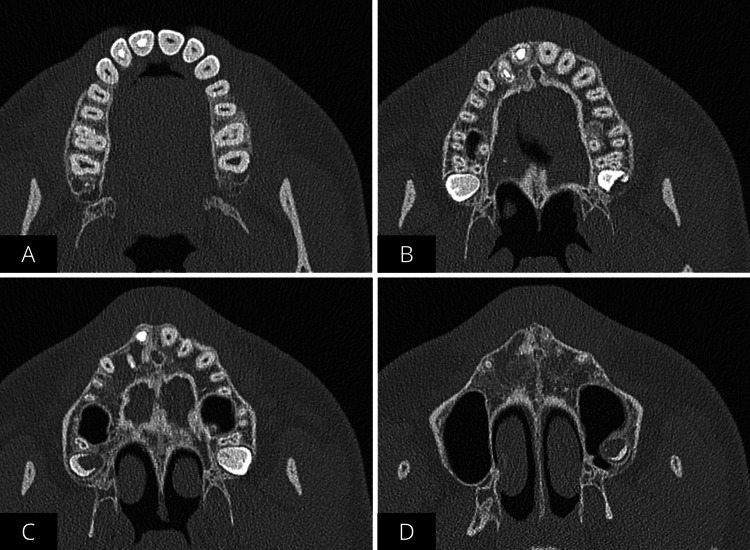
Final cone beam computed tomography showing progression of the axial view in which the complete healing of the cyst-like lesion and closure of the perforation of the buccal plate are appreciable Healing seen from the coronal aspect of elements 11 and 12 (A), middle third of elements 11 and 12 (B), apical third of elements 11 and 12 (C and D)

After one year, a follow-up control CBCT was performed, confirming complete healing (Figures 10a-10d).

The patient reported being symptom free and was able to use both teeth normally (Table 2).

**Table 2:** Clinical course of the patient CBCT: Cone beam computed tomography

Time	Event	Signs and symptoms
0	First access to the clinic, history, clinical and radiographic examination	Pain, swelling of right hemiface, abscess and swelling of labial mucosa from central incisor to canine. Pus drainage from central incisor by a colleague
0	Drainage of pus and irrigation with 10 ml of saline. Calcium hydroxide temporary medicament was placed for one month	
+1 month	Endodontic treatment and coronal reconstruction of the central incisor	Mild pain, no facial swelling, mild swelling of the labial mucosa
+3 months	First follow up: Endodontic treatment of the lateral incisor	No facial swelling, no swelling of the labial mucosa, spontaneous pain of lateral incisor, which was not responsive to thermal test (cold) and painful at percussion
+6 months	Second follow up (X-ray control-periapical radiograph)	No pain, no swelling, symptoms free, mild pain at apical palpation
+9 months	Third follow-up	No pain, no swelling, symptom-free
+12 months	Fourth follow-up (CBCT)	No pain, no swelling, symptoms free, complete healing of the lesion

## Discussion

Over the past 20 years, several studies have investigated the potential to distinguish granulomas from periapical cysts based on CBCT density.

Granulomas consist of solid soft tissue, whereas cysts are semi-solid, liquefied radiographic lesions [23]. However, clinicians cannot definitively identify the nature of the lesions before treatment: definitive diagnosis is only possible through surgical biopsy of the lesions [21]. Regarding true or ‘bay’ apical cysts, it has been demonstrated that clinically, radiographically, and histologically, there are no significant differences, except for the morphologic relationship of the cyst cavity with the root canal. Both true and bay cysts are associated with intraradicular infection and sometimes with extraradicular infection, raising doubts about the assumption that true cysts are self-sustained and not maintained by infection [6]. This supports the evidence that, regardless of whether it is a granuloma or a cyst (pocket or true), the primary treatment of choice should be an orthograde endodontic treatment [21].

Clinicians should determine treatment based on several factors, such as the extension of the lesion, relation with noble structures, origin, clinical characteristics of the lesion, age, and the systemic condition of the patient. In large lesions, endodontic treatment alone might not be efficient, and surgery might be necessary [24]. Numerous authors have stated that once the endodontic infection is eliminated, the immune system is capable of healing the lesion. Surgical intervention should only be considered for persistent cysts presenting with post-treatment apical periodontitis.

Lalonde and Luebke (1968) [25] and Bhashar (1972) [26] suggested that about 40-50% of apical lesions are radicular cysts. Given that the success rate of conventional endodontic treatment is 90%, many of these lesions respond to orthograde endodontic. The disinfection of the canal plays a fundamental role in the outcome of the therapy, and in the last 30 years, several laser technologies have been investigated as a support to enhance irrigants. The Nd:YAG laser has demonstrated its role in relation to bactericidal effects as an NaOCl supplement [13,27], and in the reduction of post-operative pain [28]. A possible alternative is the use of the diode laser in the same way as described for the Nd:YAG laser (traditional laser endodontics). The operator's choice is usually based on economic reasons, as diodes are lowest-cost devices. The diode laser can also be used in combination with a photosensitizer (aPDT), but some additional considerations must be taken into account, such as the size of the apex (it is important to avoid extrusion beyond the apex of the photosensitizer) and the possible post-treatment pigmentation [17].

Surgical approaches to cystic jaw lesions include marsupialization or enucleation [29]. In general ‘surgical endodontic treatment’, when performed using enhanced magnification, minimal root resection, ultrasonic root-end preparation, and biocompatible root-end filling materials, can achieve more than 90% of success [22]. It is important to underline that there is also an intermediate approach, which is represented by decompression of the cyst associated with traditional endodontic treatment.

Many case reports in the literature support each of these techniques. More specifically, by conducting a PubMed search on the general terms ‘management of periapical cyst - case reports’ and narrowing the field to the last 10 years (2014-2024), it is possible to find 99 results. Excluding research on dentigerous cysts, cysts related to deciduous teeth, nasopalatine duct cysts, and lateral periodontal cysts, it is possible to compare 55 articles describing four different treatment options for the resolution of radicular cyst-like lesions.

Twenty-nine articles [30-58] describe a surgical approach associated with endodontic treatment (Table 3);

**Table 3:** Surgical treatment of radicular cysts and endodontic treatment

Authors	Site	Follow-up
Sanz-Zornoza et al., 2023 [30], Guo et al., 2014 [31], Zamaliauskiene et al., 2023 [32], Kim et al., 2023 [33], Wang et al., 2022 [34], Gillner et al., 2023 [35], Borkar et al., 2016 [36]	Mandible	From 6 months to 4-5 years
Pradeep et al., 2016 [37], Elhakim et al., 2021 [38], Córdova-Malca et al., 2022 [39], Sonar et al., 2024 [40], Agrawal et al., 2023 [41], Matsuzaki et al., 2022 [42], Gómez Mireles et al., 2024 [43], Govindaraju et al., 2023 [44], Ricucci et al., 2020 [45], Manjushree et al., 2021 [46], Asgary et al., 2023 [47], Das et al., 2023 [48], Gurav et al., 2024 [49], Kadam et al., 2014 [50], Rathi et al., 2023 [51], Kaur et al., 2024 [52], Vidhale et al., 2015 [53], Salaria et al., 2020 [54], Kriplani et al., 2024 [55], Diwan et al., 2015 [56], Asgary et al., 2015 [57], Yadav et al., 2023 [58]	Maxilla	From 6 months to 4 years

five articles [59-63] describe an intermediate approach with decompression of the cyst and endodontic treatment (Table 4);

**Table 4:** Cyst decompression and endodontic treatment

Authors	Site	Follow-up
Quaresma et al., 2022 [59]	Mandible	1 year
Niavarzi et al., 2021 [60], Wang et al., 2024 [61], Cho et al., 2019 [62], Talpos-Niculescu et al., 2021 [63]	Maxilla	From 18 months to 14 years

10 articles [7,63-71] describe the conservative management of periapical cysts with endodontic treatment (Table 5);

**Table 5:** Nonsurgical management (endodontic treatment) of cyst-like lesions

Authors	Site	Follow-up
Chen et al., 2023 [64], Salaria et al., 2016 [65], Ghorbanzadeh et al., 2017 [66]	Mandible	From 1 year to 18 months
Sharma 2016 [7], Talpos-Niculescu et al., 2021 [63], Moshari et al., 2017 [67], Sood et al., 2015 [68], Keleş et al., 2015 [69], Dhillon et al., 2014 [70], Kahler 2015 [71]	Maxilla	From 1 to 2 years

and 12 articles [72-83] describe the surgical resolution of cysts without the possibility of maintaining the tooth (tooth extraction, Table 6).

**Table 6:** Surgery of cystic lesions and tooth extraction

Authors	Site	Follow-up
Bava et al., 2015 [72], Kesharwani et al., 2020 [73], Kolari et al., 2019 [74], Shivhare et al., 2016 [75], AboulHosn et al., 2019 [76], Saputra et al., 2023 [77], Noda et al., 2019 [78], Martins et al., 2015 [79]	Mandible	From 1 month to 2 years
Kolari et al., 2019 [74], Deshmukh et al., 2014 [80], Nilesh et al., 2020 [81], Hahn et al., 2019 [82], Liu et al., 2024 [83]	Maxilla	From 6 months to 2 years

Most of the case reports involve the anterior maxilla, the most commonly affected area. In most cases, follow-up periods ranged from one to two years, so even if we can judge the healing of the lesions, we do not have enough information about the long-term maintenance of the treated teeth.

## Conclusions

There are many treatment options for periapical infections, whether they are granulomas or cysts. Conservative approaches (endodontic therapy), combined approaches (endodontic therapy and surgery), and surgical approaches appear to yield similarly high success rates, as indicated by the literature analysis. 

These considerations make it clear that the choice of treatment has to be strictly linked to the individual clinical case. In the case reported here, the young age of the patient led us to consider a conservative approach as being more appropriate. The favorable clinical and radiographic outcomes of endodontic treatment in the management of large cyst-like lesions confirm that surgery can be considered as a second choice. Further studies are recommended to clarify Nd:YAG’s specific role in decontamination procedures.
